# Supraventricular Tachycardia Associated With Repeat Cesarean Section Under Spinal Anesthesia

**DOI:** 10.7759/cureus.49256

**Published:** 2023-11-22

**Authors:** Mihir Patel, Matthias Franzen, Camille D Hawkins, Matthew Brown, Samir Patel

**Affiliations:** 1 Anesthesiology, Edward Via College of Osteopathic Medicine, Monroe, USA; 2 Anesthesiology and Perioperative Medicine, OhioHealth Doctors Hospital, Columbus, USA

**Keywords:** covid-19 in pregnancy, paroxysmal supraventricular tachycardia, arrhythmia in pregnancy, carotid sinus massage, obstetric anesthesia

## Abstract

Supraventricular tachycardia (SVT) is the most common tachyarrhythmia of pregnancy. Catecholamine surges, the use of vasoactive agents during delivery, and increased cardiac output during pregnancy are the most common contributing factors to developing SVT. SVT is usually benign in presentation but can lead to more serious arrhythmias in patients with a history of mitral stenosis secondary to rheumatic heart disease. When an SVT is detected, organic heart causes should be ruled out first. Symptoms of SVT include shortness of breath, palpitations, syncope, sweating, chest pain, and dizziness. In patients who are refractory to pharmacologic management and hemodynamically unstable, electrical cardioversion has proven to be efficacious and safe in all trimesters. The initial treatment for hemodynamically stable patients is to attempt vagal maneuvers, such as carotid sinus massage or Valsalva maneuver. If the SVT does not convert to normal sinus rhythm, treatment with adenosine or beta-blockers may be initiated. Treatment with atenolol and verapamil should be avoided due to their teratogenic effects.

## Introduction

Supraventricular tachycardia (SVT) is an irregularly fast heartbeat that primarily affects the atrial chambers of the heart. Pregnant women are more likely to experience arrhythmias due to the physiologic increase in blood volume, which, in return, increases atrial stretching. SVT is the most common sustained arrhythmia to occur during pregnancy with a prevalence of 24 per 100,000 hospital admissions. Approximately about 20% of patients with pre-existing SVT will experience symptomatic exacerbations during pregnancy [1]. In most cases of SVT during pregnancy, there is no history of heart disease, though potential risk factors include congenital or structural heart disease, and hyperthyroidism [2]. Common triggers of SVT during labor include catecholamine release, electrolyte disturbances, and vasopressors administered to treat post-epidural hypotension [3]. Parturients experiencing arrhythmias intrapartum are at a higher risk of perioperative complications. Prompt identification and treatment are necessary to prevent morbidity and mortality [4]. Here we discuss a case of SVT in a parturient during cesarean section. 

## Case presentation

**Figure 1:**
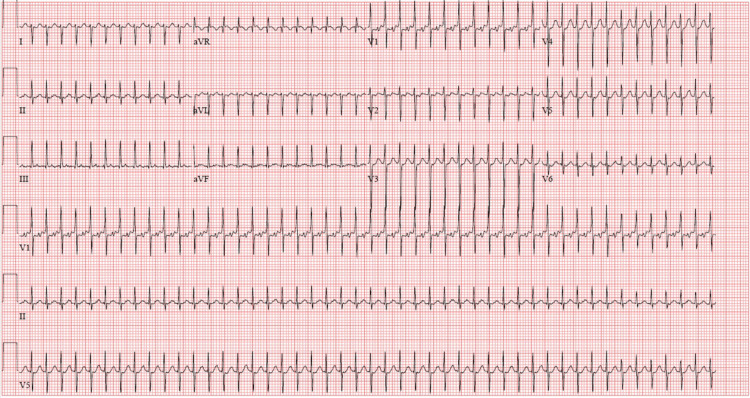
Patient's EKG obtained during surgery, which shows supraventricular tachycardia

A 31-year-old gravida 5 para 2 female, with no significant past medical history, presented for a scheduled repeat cesarean section and tubal ligation under spinal anesthesia. Her past medical history includes prior postpartum hemorrhage, a history of blood transfusion, headaches, anxiety, and anemia. Spinal anesthesia with 2mL 0.75% bupivacaine in dextrose with 200mcg morphine was administered at vertebral level L3-L4. The patient was placed on 3 liters of oxygen delivered via nasal cannula and laid in the supine position with left uterine displacement and standard ASA monitoring guidelines were applied. After the surgery was initiated, the patient began having supraventricular tachycardia with a heart rate of 145-155 bpm, as shown in Figure 1, that was unresponsive to 100mg esmolol and 10mg metoprolol; however, she remained hemodynamically stable. The patient's basic metabolic profile (BMP) and complete blood count (CBC) were within normal limits, so there was no identifiable organic cause of the SVT. The patient was unable to participate in vagal maneuvers, so a carotid massage was initiated which abated the SVT. While the baby was being delivered, the parturient's heart rate returned to 100 bpm. The patient tolerated the remainder of the surgery without any further episodes of arrhythmias.

## Discussion

The management of SVT in pregnancy can present unique challenges; physiologic changes in pregnancy lead to an increase in oxygen demand relative to increased cardiac output along with hormonal changes and catecholamine release. Anesthetic involvement must be considered since spinal anesthesia can potentiate sympathetic blockade and hypotension, both of which could exacerbate an SVT [2]. In a new onset SVT, the Advanced Cardiovascular Life Support (ACLS) algorithm should be followed for a hemodynamically stable patient with narrow complex tachycardias. Treatments include adenosine, beta-blockers, calcium channel blockers, and vagal maneuvers (eg., Valsalva and carotid body massage). The most common clinical manifestations of SVT include palpitations, shortness of breath, sweating, syncope, chest pain, and dizziness [4,5]. Our patient admitted feeling like her heart was racing but remained hemodynamically stable. She did not respond to the treatment of beta blockers, however, the SVT was abated during carotid body massage and as the baby was delivered, though the actual reason for the resolution is difficult to ascertain.

The vagal maneuver is the most appropriate initial intervention for the termination of an SVT [6]. This is generally well tolerated in the first trimester and many times the SVT will resolve [1]. If the vagal maneuver does not resolve the SVT, pharmacologic interventions must be explored. The first-line pharmacologic agent that can be used in the first trimester is adenosine. Adenosine is considered safe in pregnancy for SVT since it has a very short half-life of only 10 seconds, making it highly unlikely to enter fetal circulation [7]. Patients who are refractory to adenosine can be treated with second-line agents, which include beta-adrenergic blockers such as propranolol and metoprolol. Digoxin can also be used if beta blockers and adenosine have not been effective, but extensive monitoring may be required with the use of this drug [8,9]. Dioxin is a cardiac glycoside that can readily cross the placenta and equilibrates between maternal and fetal serum concentration quickly. It affects cardiac rate and rhythm by increasing vagal activity in the central nervous system (CNS), resulting in slowed conduction in the atrioventricular(AV) node.

Digoxin also directly inhibits the sodium-potassium adenosine triphosphatase and indirectly inhibits the sodium-calcium exchanger, leading to increased calcium concentrations in cardiac myocytes causing a positive inotropic effect. Tight monitoring of serum digoxin levels is required due to the narrow therapeutic window. Vomiting should be considered an early sign of toxicity, but other possible side effects may include nausea, diarrhea, blurry vision, and fetal complications such as fetal arrhythmias [10,11]. In patients where no pharmacologic agents are useful in the termination of the SVT and if they start to become unstable, synchronized cardioversion has been shown to be safe and efficacious throughout pregnancy. During the course of pregnancy, medications that can be used during the second and third trimesters include atenolol and verapamil, both of which are contraindicated during the first trimester. Atenolol can cause intrauterine growth restrictions, leading to low birth weight, and should only be considered in life-threatening situations. Verapamil can abate the SVT, but it can also enter fetal circulation, leading to fetal arrhythmias and heart blocks [1,3]. Amiodarone is known to cause fetal hypothyroidism, growth retardation, and prematurity [12].

For our patient, a 12-lead EKG was obtained postoperatively demonstrating sinus tachycardia. The postoperative course was complicated by acute blood loss anemia with a starting hemoglobin of 9.3 g/dl dropping to 6.3 g/dl. The acute blood loss was enough to trigger a cardiac arrhythmia as well. She required a blood transfusion with 1 unit of packed red blood cells. She then developed a fever of unknown origin. The blood cultures were negative, so the most probable source of the fever was her blood transfusion. She was discharged home on postoperative day four without any repeat occurrences of cardiac arrhythmia.

## Conclusions

SVT in pregnancy is not an uncommon occurrence. Causes of SVT during delivery could be precipitated by the use of vasopressors, catecholamine surges, and physiologic increases in cardiac output at term. If new onset narrow complex SVT occurs, the main treatment protocol is to follow the adult tachycardia with a pulse algorithm given by the American Heart Association (AHA). This case presentation highlights the importance of proper identification and management of pregnant patients with new onset SVT; the case illustrates successive interventions that are appropriate to follow. If there is a lack of response with vagal maneuvers or carotid sinus massage, the next best step would be ablation of SVT with adenosine followed by beta-blocker, propranolol, or metoprolol. Medications that can be used during pregnancy during the second and third trimester include atenolol and verapamil, both of which have a Class C FDA rating. Digoxin could be a final consideration although specialized supervision may be required. Ultimately, if medical interventions are unsuccessful or the patient becomes hemodynamically unstable, synchronized cardioversion is safe and can be used in any trimester.
